# Effect of the Combining Corn Steep Liquor and Urea Pre-treatment on Biodegradation and Hydrolysis of Rice Straw

**DOI:** 10.3389/fmicb.2022.916195

**Published:** 2022-07-13

**Authors:** Yulin Ma, Xu Chen, Muhammad Zahoor Khan, Jianxin Xiao, Gibson Maswayi Alugongo, Shuai Liu, Jingjun Wang, Zhijun Cao

**Affiliations:** ^1^State Key Laboratory of Animal Nutrition, College of Animal Science and Technology, China Agricultural University, Beijing, China; ^2^Department of Animal Sciences, Faculty of Veterinary and Animal Sciences, University of Agriculture, Dera Ismail Khan, Pakistan

**Keywords:** rice straw, corn steep liquor, microbial colonization, urea-pre-treatment, enzymatic hydrolysis

## Abstract

A novel pre-treatment using corn steep liquor (CSL) and urea was developed to enhance the enzymatic saccharification and degradability of rice straw (RS). We used RS (1) without (Con) or with additives of (2) 5% urea (U), (3) 9% CSL and 2.5% urea (CU), and (4) 9% CSL and 5% urea (C5U). The result showed that the water-soluble carbohydrate (WSC) conversion of RS reached 69.32% after C5U pre-treatment. Scanning electron microscopy (SEM), Fourier transform infrared (FTIR) spectroscopy, and X-ray diffraction analysis (XRD) confirmed that the surface of pre-treated RS exposed more cellulose and hemicellulose due to the disruption of the resistant structure of lignocellulose. Pre-treated RS significantly decreased neutral detergent fiber (NDF) and acid detergent fiber (ADF) contents and increased crude protein (CP) content, microbial colonization, and induction of *Carnobacterium* and *Staphylococcus* attachment. Altogether, we concluded that pre-treatment of a combination of CSL and urea has the potential to improve the nutritive value of RS.

## Introduction

Lignocellulosic biomass is one of the most abundant renewable resources globally, and converting it into biofuels is an effective way to alleviate energy shortages (Zheng et al., 2014). Rice straw (RS) is mainly composed of cellulose, hemicellulose, and lignin and is one of the main lignocellulosic biomass in the world (Tsapekos et al., 2017). However, RS's highly complex and resistant rigid cellulose-hemicellulose-lignin structure could increase the difficulty of biodegrading and hydrolyzing the RS, which was regarded as rate-determining steps in anaerobic digestion (Martin-Ryals et al., 2015; Yu et al., 2016). Thus, a large amount of lignocellulose cannot be converted and utilized in eco-friendly ways, and burning RS contributes a lot to environmental pollution (Ozbayram et al., 2018). Recently, different pre-treatments have been applied to improve the degradation of lignocellulose in anaerobic digestion, including physical (Wang et al., 2019), chemical (Fang et al., 2020), and biological (Takizawa et al., 2019) approaches. However, these pre-treatments usually take issues with high cost, low efficiency, and insufficient combined utilization of agricultural by-products (Ali et al., 2021; Sun et al., 2021), and a higher potency and an economic approach are in urgent need.

Urea pre-treatment has been recognized as one of the commonly mature methods, which has been widely used in the pre-treatment of agricultural by-products, considering the merits of breaking the lingo-cellulosic bonds effectively, improving the nutrition value of straw, low-costing, approximately 449.7 $/tone, and easy-accessing (Ribeiro et al., 2020; Yuan et al., 2020); Nevertheless, urea pre-treatment often releases a large amount of ammonia volatilizes into the atmosphere during the processing, only 30–35% of the nitrogen are retained in the straw (Sarwar et al., 2003). By taking into account the pollution and inefficiently utilized nitrogen resulting from urea pre-treatment, researchers tried to focus on fixing nitrogen with HCL and H_2_SO_4_ (Elseed, 2015; Wu et al., 2021; Zhang et al., 2021), but these used chemicals aroused the discussion of safety, operation, and high costs. Corn steep liquor (CSL), an acidoid, can fix nitrogen; it is also a by-product of corn starch processing, which makes it easy to access and friendly to the environment (Qamar et al., 2015), while CSL was low-costing, ~44.9 $/tone. It was reported that 9% CSL treated wheat straw could improve Buffalo bulls' growth performance and rumen fermentation (Nisa et al., 2006), but the mechanism is still unknown. Therefore, this study will explore the effect and mechanism of the combination of CSL and urea pre-treatment on RS.

On account of the rumen, microbial communities attached to feed particles are a key step in the rumen fermentation and digestion, and microbial compositions (such as *Fibrobacter succinogenes, Ruminococcus flavefaciens*, and *Ruminococcus albus*) were used frequently as an evaluation index to analyze the degradation of feed (Mcallister et al., 1994). Studies found that neutral detergent fiber (NDF), acid detergent fiber (ADF), and crude protein (CP) could affect microbial colonization; for example, cellulolytic bacteria (*Fibrobacter, Ruminococcus*, and *Butyrivibrio)* tended to attach to feeds with high NDF (Liu et al., 2016). However, few studies have reported the effect of pre-treated RS on the adhesion of microbes in the rumen. Most studies only focused on improving the raw feed materials (Gharechahi et al., 2020), not to mention the diversity and mechanism of rumen microbes. Additionally, understanding the dynamics of bacteria attached to urea pre-treated RS may provide opportunities to improve the nutrient efficiency of low-quality forages and the manipulation of rumen microbial communities. Nevertheless, very limited data are available on the diversity of rumen bacteria, their attachment preferences, and their degradation in RS by urea pre-treated RS.

Given all this, our study aimed to explore the effects of combining CSL and urea pre-treatment on chemical composition, physicochemical, enzymatic hydrolysis, and attachment properties of rumen microbes of RS, which will not only uncover the dynamics and mechanism of how CSL and urea pre-treatment boost the rumen microbes to attach to the RS but also will provide a new sight and useful mode of RS fodder processing, as well as looking forward to its developed prospect.

## Materials and Methods

### Ethical Statement

The *in situ* experimental procedure was approved by the Ethical Committee of the College of Animal Science and Technology of China Agricultural University (protocol number: 2013-5-LZ).

### Anaerobic CSL and Urea Treatment

The RS was randomly collected from the Suburb of Gushi County, Henan Province of China. CSL and urea were provided by Henan Yuyao New Medicine Co. LTD and Henan Hand-in-Hand Fertilizer Co. LTD (Henan, China), respectively.

The 500 g of RS were weighted and chopped into 2–3 cm lengths and stored in laboratory polyethylene 25 × 35 cm sterile bags purchased from Beijing Shengya Yuda Biological Technology Co., Ltd. (Beijing, China), and a total of 60 bags of RS were prepared. These bags were pre-treated into four different approaches based on dry matter (DM): (1) no pre-treatment for control group (Con), (2) 5% urea group (U), (3) 9% CSL + 2.5% urea group (CU), and (4) 9% CSL+ 5% urea group (C5U), and each approach contained 15 bags. The DM contents of all groups were adjusted to 45% by adding water. These bags were sealed using a food vacuum sealing machine (Konka KZ-ZK007; Dongguan Yijian Packaging Machinery Co. Ltd, Dongguan, China) and stored at room temperature (25 ± 4°C) for 15 days. Later, all pre-treated RSs were sampled for chemical analysis and enzymatic hydrolysis by oven-dried (65°C for 48 h) and ground in a hammer mill to pass a 1 mm sieve, evaluating structural changes, as well as a profile of the microbial communities attached to the pre-treated RS, and each sample was tested in triplicate.

### Chemical Composition Analysis

The DM, CP, and crude ash content (Ash) of the RS samples were determined according to the method described by AOAC (1984). The NDF and ADF were measured using an A2000 Fiber Analyzer (ANKOM Technology Corp., Macedon, NY, USA) following the method adopted from the previous study (Van Soest et al., 1991).

### Structural Analysis

ASU 3500 (Japan) scanning electron microscopy (SEM) was used to observe any morphological changes in the RS biomass before and after CSL and urea pre-treatment at a magnification of 1,500. Prior to imaging, the RS samples were sputter-coated with platinum to make the materials conductive.

The Fourier-transform infrared (FTIR) spectra of RS before and after CSL and urea pre-treatment were recorded using a Bruker Vertex 70 FTIR spectrophotometer (Bruker, Ettlingen, Germany) equipped with an RT-DLaTGS detector at 4,000–1,000 cm^−1^ with a resolution of 4 cm^−1^ and 16 scans per sample. Prior to scanning, fine ground samples (200 meshes; 1.0 mg) were mixed with KBr (50 mg) and pressed into a pellet for analysis at 1 MPa of pressure. Before data collection, background scanning was performed for correction.

X-ray diffraction (XRD) was conducted using a Siemens D-5000 diffractometer (Bruker, Ettlingen, Germany), and Cu-K radiation was generated at 40 kV and 20 mA. Samples were scanned from 3 to 40° with a step size of 0.02 and 3 s per step. The cellulose crystallinity index (CrI) was calculated using the following formula (Segal et al., 1959):


(1) $$\begin{array}{r} \text{CrI}=\;\left({\text{I}}_{002}-I_{\text{am}}\right)/{\text{I}}_{002} \end{array}$$

where *I*_002_ is the scattered intensity at the main peak for cellulose type I; *I*_am_ is the scattered intensity due to the amorphous portion evaluated as the minimum intensity between the main and secondary peaks.

### Enzymatic Hydrolysis

Cellulase mixture SAE0020 (Sigma) was used for enzymatic hydrolysis experiments, in which enzymatic hydrolysis activity was 120 FPU/ml, and commercial β-glucosidase preparation activity was 30 CBU/mg. First, enzymatic hydrolysis was carried out in a 125 ml Erlenmeyer flask with a solid content of 5% (w/v), an enzyme content of 20 FPU/g, and 15 CBU/g DM. Notably, 50 mM sodium citrate buffer and incubation thermostat air bath shaker set at 50° and 180 rpm for 72 h at pH 4.8 were used in the current experiment. To prevent microbial contamination, 0.02% (v/v) hydrolase was added before Proclin. The enzyme blank (without substrate) was run in parallel with other samples. A 1 ml sample with a knife-tip pipette was taken and stored for 72 h incubation. Then, the enzymatic hydrolysate was centrifuged at 3,000 *g* for 5 min, and the yield of water-soluble carbohydrates (WSCs) was measured together with the supernatant.

### *In situ* Rumen Incubation

The dried RS samples were milled through a 2 mm sieve, and 5 g of sample was weighed into nylon bags (8 × 16 cm, 50 μm pore size) in six repetitions. Additionally, these samples were incubated for 0.5, 4, 12, and 24 h in three cannulated Holstein Friesian cows (each cow had two repeats of each sample, 32 bags per cow). The diet of cows is shown in Supplementary Table 1.

After removing the bags at each time point, the bags were washed gently with phosphate-buffered saline (PBS, pH 7.4) three times to remove liquid-borne and loosely attached microbiota. The bags were finally hand-squeezed using sterile gloves to remove excess water. The samples were then transferred in liquid nitrogen to the laboratory and stored at −80°C for subsequent DNA extraction. The colonization of ruminal microbes on the RS was quantified by real-time polymerase chain reaction (PCR). Primer pairs for total bacteria were Eub338F (ACTCCTACGGGAGGCAGCAG) and Eub806R (GGACTACHVGGGTWTCTAAT).

### The Colonization of Ruminal Microbial Structure Analysis

The EZNA stool DNA Kit (Omega Biotek, Norcross, GA, US) was used to extract microbial DNA. The Majorbio Cloud Platform (www.Majorbio.com) was used to analyze the high-throughput sequencing. The V3-V4 variable region of the 16S rDNA was targeted using primers Eub338F (ACTCCTACGGGAGGCAGCAG) and Eub806R (GGACTACHVGGGTWTCTAAT). The PCR reactions consisted of an initial denaturation at 95°C for 5 min followed by 35 cycles at 95°C for 30 s, 58°C for 30 s, 72°C for 1 min, and a final extension at 72°C for 5 min. The reactions were performed in a 20 μl mixture containing 10 μl of 2X Taq Plus Master Mix, 0.8 μl of each primer (5 μM), 7.4 μl of ddH_2_O, and 1 μl of each reaction that was used as a template for PCR. Thus, each sample was performed in triplicate of PCR reactions.

### Statistical Analysis

All the data were analyzed using the IBM SPSS Statistics 24 (SPSS Inc., Chicago, IL, USA). One-way ANOVA analysis was performed to examine the effect of CSL and urea pre-treatment on the chemical composition of RS. In addition, the Duncan multiple comparison method was carried out to compare the differences between the means; *P* < 0.05 was used to show significance levels. The DNA sequencing data were analyzed on a free online platform of Majorbio tools https://cloud.majorbio.com/page/project/p.html.

## Results and Discussion

### Chemical Composition Changes of RS With CSL and Urea Pre-treatment

In terms of DM content, no significant difference has been observed between the three urea pre-treated groups and the control group (*P* = 0.890, Table 1), but the content of CP in U was significantly increased (*P* < 0.05), in comparison to that in Con. Generally, a higher CP level was beneficial for higher DM digestibility (Milis Ch, 2007), and the urea pre-treatment could effectively increase the CP content in the RS (Salami et al., 2021), which is why the urea pre-treatment has widely been used in production; however, a large percentage of 65–70% nitrogen was wasted as releasing into the atmosphere (Sarwar et al., 2005). Notably, our results demonstrated that the CP contents of CU and C5U were significantly higher than that in U (*P* < 0.05), indicating that CSL had promoted nitrogen fixation in the RS. Similar changes in CP contents were reported in wheat straw pre-treated with CSL (Nisa et al., 2006). This means that CSL has the potential to improve the nutritional value of low-quality roughages.

**Table 1:** Effect of corn steep liquor (CSL) and urea pre-treatment on the chemical composition of anaerobically stored rice straw (RS).

**Items**	**Con**	**U**	**CU**	**C5U**	**SEM**	***P*-Value**
DM, %	95.65	95.83	95.84	95.75	0.19	0.890
CP, %DM	5.05^c^	9.01^b^	12.04^a^	12.13^a^	0.02	<0.001
NDF, %DM	66.38^a^	65.45^b^	63.24^c^	62.12^d^	0.01	<0.001
ADF, %DM	56.11^a^	53.24^b^	49.26^c^	48.03^d^	0.05	<0.001
Ash, %DM	13.86^b^	12.90^d^	14.25^a^	13.34^c^	0.04	<0.001

*Different superscript letters a, b, c, and d indicate significantly different values (P < 0.05) across rows, and the same letters indicate insignificant differences (P > 0.05). Con, without additive control; U, 5% urea; CU, 9% CSL + 2.5% urea; C5U, 9% CSL + 5% urea; DM, dry matter (the dry matter content is calculated based on air-drying the sample); CP, crude protein; NDF, neutral detergent fiber; ADF, acid detergent fiber*.

The high NDF and ADF contents can significantly reduce the utilization of RS (Passetti et al., 2020). Eisenhuber et al. (2013) found that urea pre-treatment could cause the breakdown of the complex structure of RS lignocellulose and dissolve a part of cellulose and hemicelluloses (Eisenhuber et al., 2013). Our study obtained the same conclusion, and according to the finding, the contents of NDF and ADF in U were observed significantly lower than that in Con (*P* < 0.05). When urea is dissolved in water and became alkaline, the alkaline could contribute to the reducing content of NDF and ADF in RS by destroying the lignocellulose structure of RS (Lam et al., 2001; Xu et al., 2012; Li et al., 2014). In addition, our study also determined that the breakdown influence of urea pre-treatment on RS will increase with the increase of urea content, based on our result of NDF and ADF contents of the C5U and CU group that was significantly lower than U (*P* < 0.05). This may be due to the nitrogen fixation of CSL, which increases the alkalinity and destroys the lignocellulose structure of RS. Previous studies have documented that alkali pre-treatment destroys the lignocellulosic structure of corn bran (Yue et al., 2022).

### Structural Changes of RS With CSL and Urea Pretreatment

**Figure 1:**
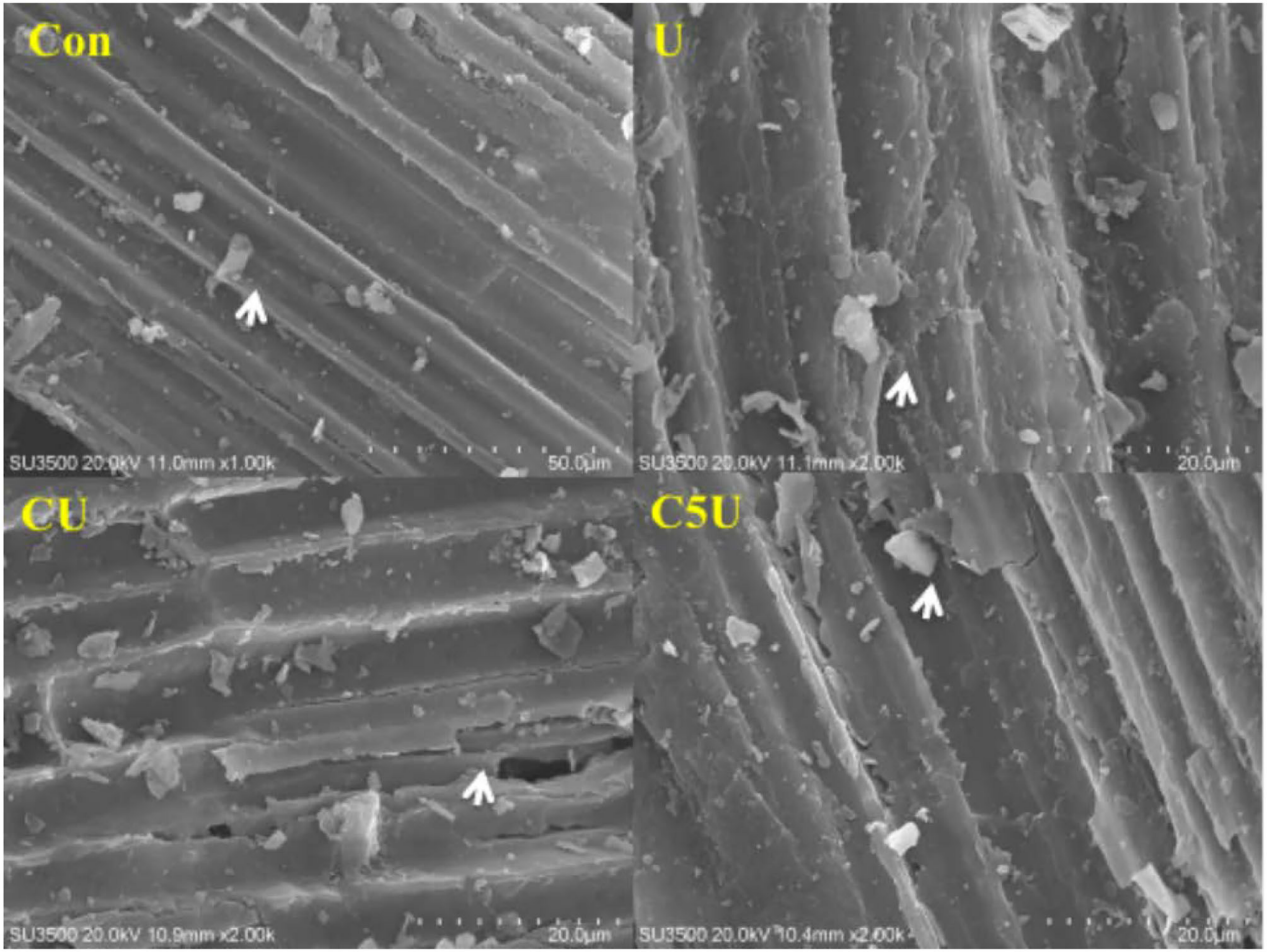
Scanning electron microscopy (SEM) images of biomass residues obtained from pre-treatment with corn steep liquor (CSL) and urea. Con, without additive control; U, 5% urea; CU, 9% CSL + 2.5% urea; C5U, 9% CSL+ 5% urea. Sample (U, CU, and C5U) showing a coarse surface indicated as an arrow, and sample Con displaying a flat face.

To describe the morphology changes of CSL and urea pre-treated RS, our study investigated the RS structure by SEM (Figure 1). The untreated RS exhibited surface structures that were non-decayed and smooth; these might hinder enzyme or rumen bacterial attachments. In addition, the surface of CU and C5U had more serious damage than that in U (Figure 1), which proved that the combination of CSL and urea pre-treated RS might improve the digestibility and rumen fermentation, due to which CSL could effectively fix nitrogen and enhance the alkaline function of urea pre-treatment to destroy the surface structure of RS. The SEM images visually showed the higher lignocellulolytic degradation potential of microbial. These results also corroborate the variation in NDF and ADF contents as depicted in Table 1. The structural changes would increase the biomass's surface area, which allows for greater accessibility to an enzyme or microbial attachment. Previous studies have proved that rumen microbial colonization on the feed surface is crucial for biomass degradation, and there is a positive correlation between the amount of colonization and degradation performance (Gharechahi et al., 2020; Vahidi et al., 2021).

**Figure 2:**
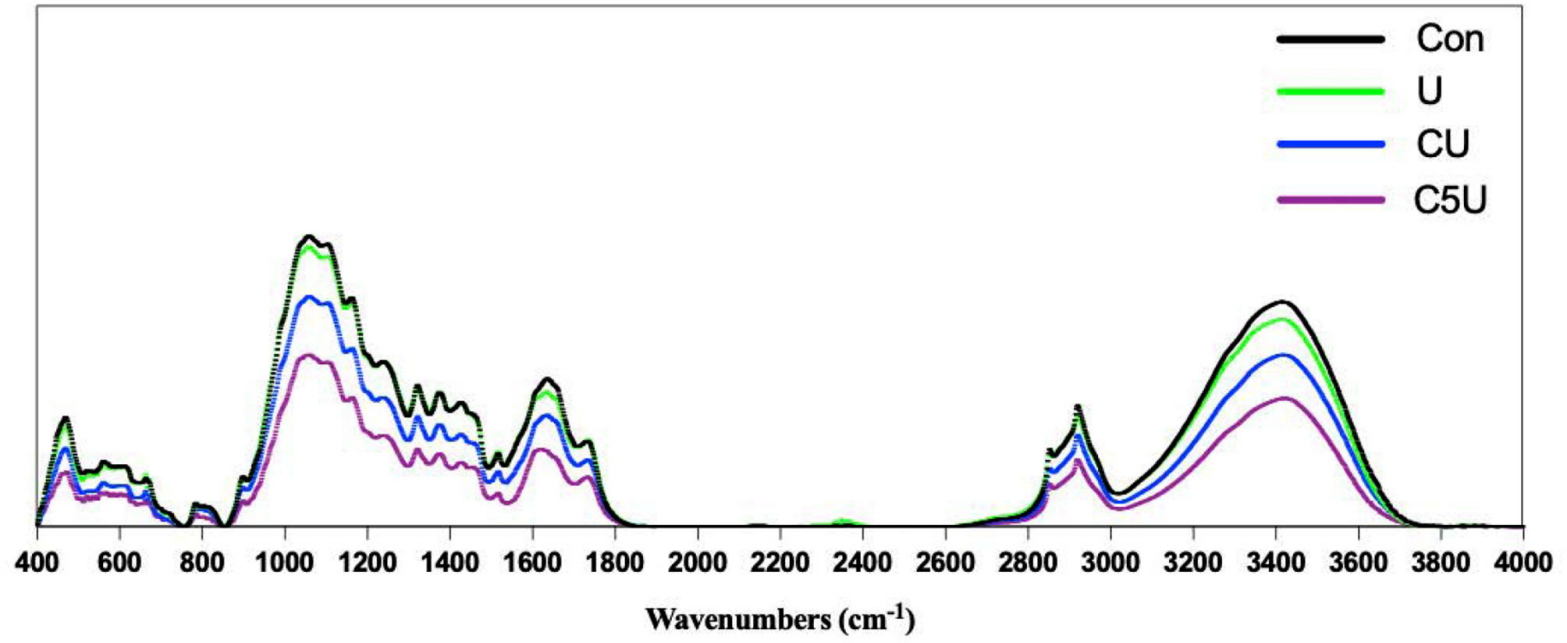
Fourier-transform infrared (FTIR) spectroscopy of rice straw (RS) after CSL and urea pre-treatment. Con, without additive control; U, 5% urea; CU, 9% CSL+ 2.5% urea; C5U, 9% CSL+ 5% urea.

We further explored the changes in the chemical properties of RS after CSL and urea-pre-treated by FTIR (Figure 2). In FTIR spectrometry, the structural changes in cellulose, hemicellulose, or lignin are reflected in characteristic absorbance bands. After CSL and urea-pre-treated, the bands at 1,098 cm^−1^ due to C–OH and C–O–C stretching vibrations increased (Liang et al., 2018), suggesting increased amounts of exposed cellulose from the reduction of hydrogen bonds and erosion of CSL and urea pre-treated surfaces. Characteristic peaks of cellulose and hemicellulose at the absorbance of 1,375 cm^−1^ increased compared with that of Con, indicating that hemicellulose and other carbohydrates have been hydrolyzed. The relative intensity in the aromatic ring observed from lignin at 1,515 and 1,427 cm^−1^ increased after CSL and urea pre-treatment (CU and C5U). This could be explained by an increased relative amount of lignin due to decreased hemicellulose content. The observed structural changes in RS enabled to access and digestion by anaerobic microbes, which enhanced the degradability of the RS (Zhang et al., 2015).

**Figure 3:**
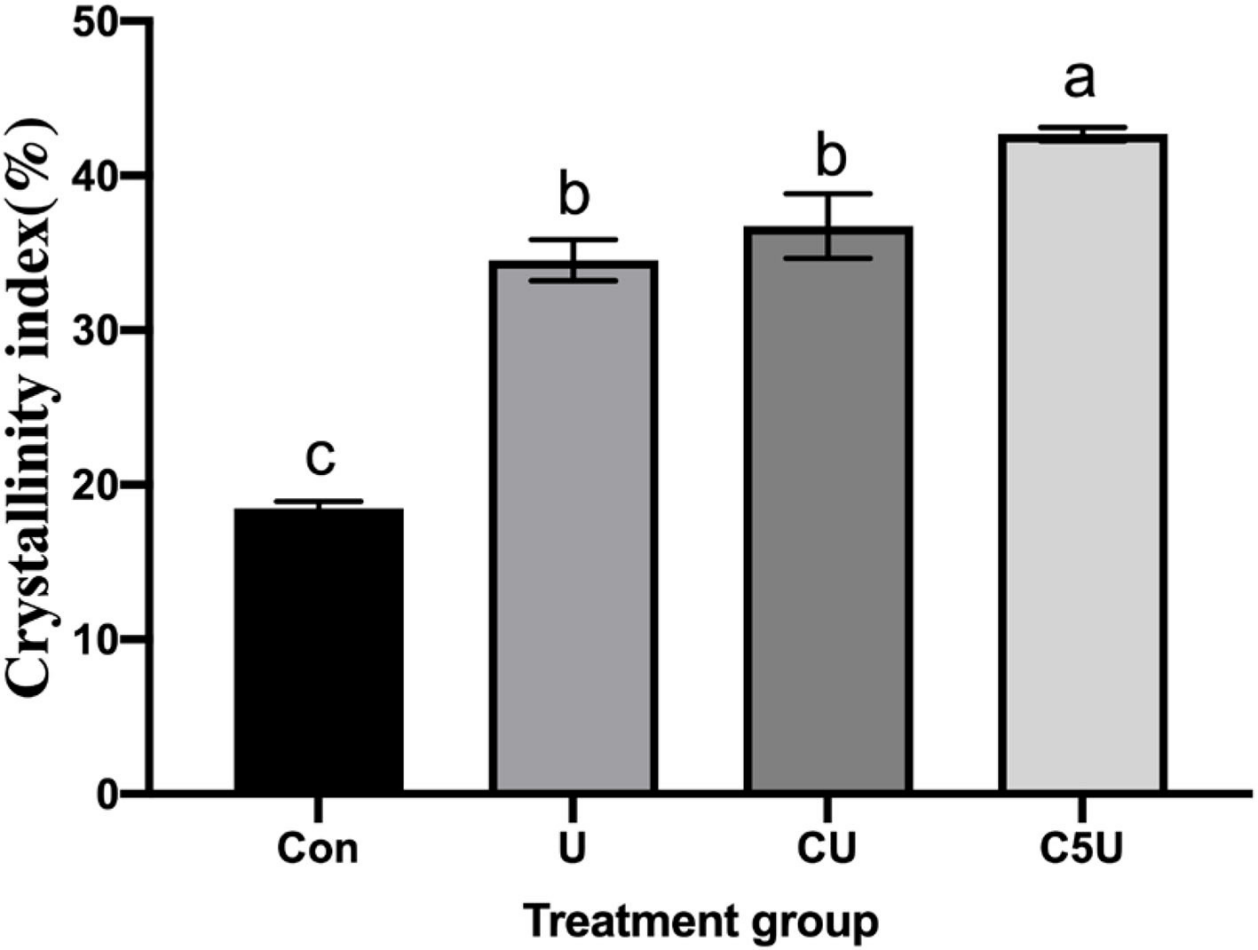
Effect of CSL and urea pre-treatment on the cellulose crystalline index (CrI, %) of RS. Con, without additive control; U, 5% urea; CU, 9% CSL + 2.5% urea; C5U, 9% CSL + 5% urea. Different superscript letters a, b, c, and d indicate significantly different values (*P* < 0.05) in different groups, and the same or no letters indicate insignificant differences (*P* > 0.05).

To further explore the effect of CSL and urea-pre-treated on the cellulose structure, we analyzed the changes of the straw CrI by XRD. We found that the CrI of U, CU, and C5U increased obviously (*P* < 0.05) compared with the Con group (Figure 3). Notably, the CrI of straw has a significant correlation with degradation. The higher the CrI, the lower the degradation rate (He et al., 2019). But this is not absolute, another study also reported that the CrI of straw increased by urea-treated (Shafiei et al., 2015; Karimi and Taherzadeh, 2016; Pan et al., 2017). The reasons for the increase of CrI after CSL and urea-pre-treated in this study may be as follows. (1) CSL and urea-pre-treated largely removed amorphous substances in RS (Donaldson, 2007), such as amorphous cellulose and noncellulose polymers, leading to an increase in cellulose CrI (Naoki et al., 1997). (2) Degradation of amorphous hemicellulose in CSL and urea-pre-treated RS (Huang et al., 2022). (3) The dissolution of considerable amorphous cellulose strengthened the hydrogen bonds among cellulose molecules, forming more contact macromolecule chains, consequently increasing the CrI of α-cellulose (Naoki et al., 1997). Although it has been documented that CrI is an important characteristic of lignocelluloses in hydrolysis, it still cannot be considered the sole effective factor.

### Enzymatic Hydrolysis of RS After CSL and Urea Pre-treatment

**Figure 4:**
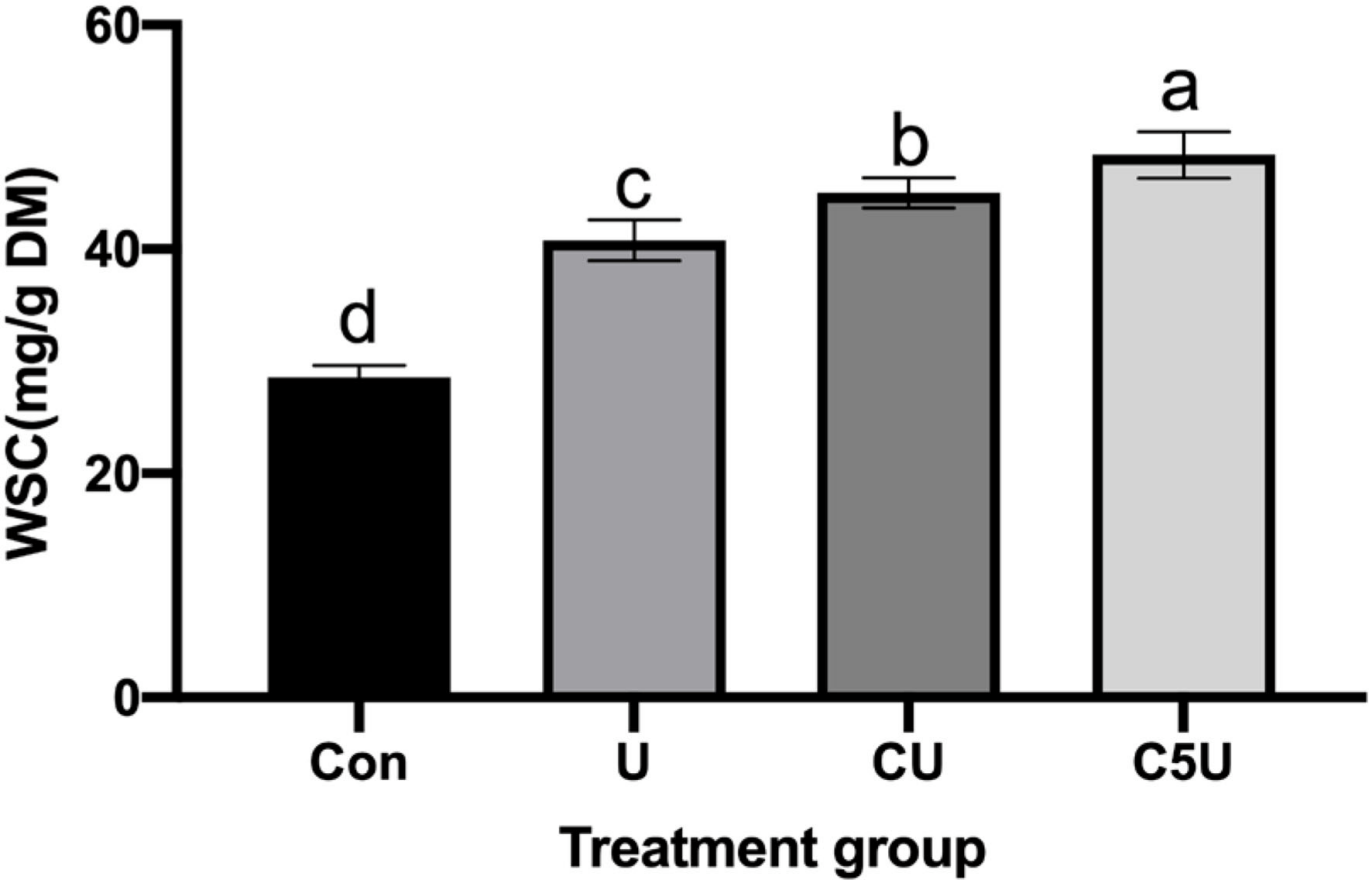
Effect of CSL and urea pre-treatment on the enzymatic water soluble carbohydrate (WSC) yield of RS. Con, without additive control; U, 5% urea; CU, 9% CSL + 2.5% urea; C5U, 9% CSL + 5% urea. Different superscript letters a, b, c, and d indicate significantly different values (*P* < 0.05) in different groups, and the same or no letters indicate insignificant differences (*P* > 0.05).

Direct enzymatic hydrolysis of lignocellulosic materials could be an indicator of biomass utilization efficiency during microbial fermentation (Fidio et al., 2020). Figure 4 illustrates the WSC yield of CSL and urea-pre-treated. As excepted, the sturdy structure of lignocellulose hindered enzymatic hydrolysis in untreated RS, and the CSL and urea pre-treatment clearly improved the availability of carbohydrates for enzymatic hydrolysis, evidenced by higher WSC released from U (40.79 mg/g), CU (45.03 mg/g), and C5U compared with the Con (28.59 mg/g) group. Notably, such an increase in WSC could be attributed to the decrease in fiber fractions and alteration in cell wall structural matrix, which was also consistent with our results of structural analysis (Figures 1, 2) that all urea-pre-treated groups had destroyed the straw structure. Thus, the RS pre-treated with CSL and urea could provide more nutrients for rumen microbial to improve the production performance of ruminants.

### RS Attached Bacterial Density After CSL and Urea Pretreatment

Total bacterial populations in the RS samples were estimated by real-time PCR analysis. Results showed that the CSL and urea pre-treatment, incubation time, and their interactions could significantly (*P* < 0.001) affect the copy number of bacterial 16S rRNA genes determined on RS (Table 2), and the greatest bacterial numbers were observed at 24 h (*P* < 0.001). In addition, our study observed that bacteria tend to attach to RS from C5U, CU, and U, instead of Con (*P* < 0.001), which showed the positive impact of microbial colonization on the surface of RS of CSL and urea pre-treatment. Notably, the colonization of microbes on fibrous feed particles was affected by the WSC and structure characteristics of the substrate (Miron et al., 2001). Apparently, the CSL and urea pre-treatment increased the RS surface area that microbes could colonize.

**Table 2:** Effect of CSL and urea pre-treatment on the particle attached bacterial density in the rumen.

**Items**	**Treatment**				**SEM**	**Hour**				**SEM**	***P*** **-value**		
	**Con**	**U**	**CU**	**C5U**		**0.5**	**4**	**12**	**24**		**T**	**H**	**T × H**
MC(log_10_/g)	9.61^d^	9.85^b^	9.73^c^	9.96^a^	0.14	8.60^d^	9.89^c^	10.21^b^	10.46^a^	0.08	<0.001	<0.001	<0.001

*Different superscript letters a, b, c, and d indicate significantly different values (P < 0.05) across rows, and the same or no letters indicate insignificant differences (P > 0.05). MC, microbial colonization (log_10_ gene copies/g of undigested RS). Con, without additive control; U, 5% urea; CU, 9% CSL+ 2.5% urea; C5U, 9% CSL+ 5% urea. SEM, standard error of means. T, treatment; H, hour; T × H, treatment × hour*.

### Effect of CSL and Urea Pre-treatment on Bacterial Community Structure

**Figure 5:**
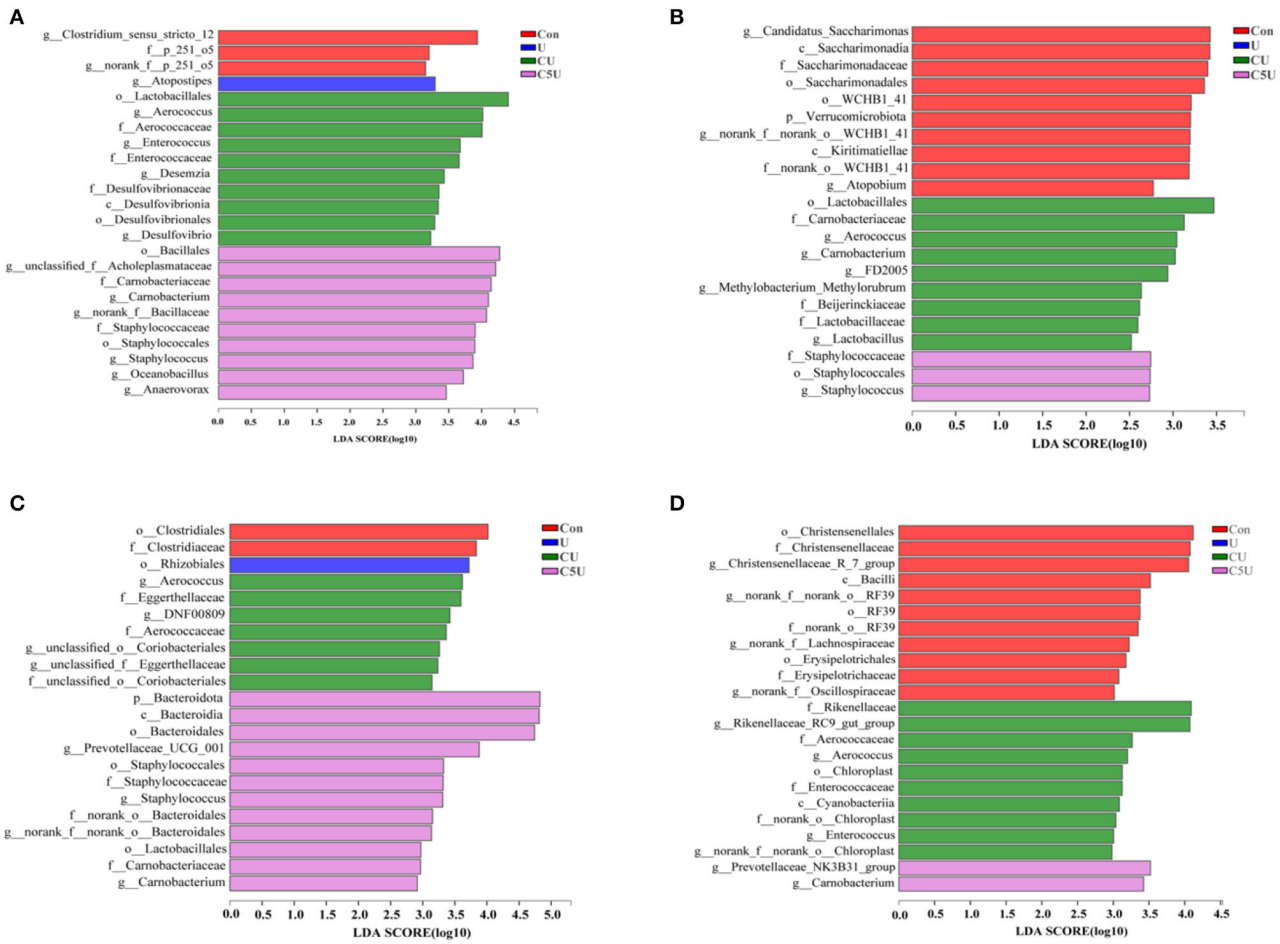
LEfSe analyses of the bacterial community shift to RS after 0.5 h **(A)**, 4 h **(B)**, 12 h **(C)**, and 24 h **(D)** within the rumen. Con, without additive control; U, 5% urea; CU, 9% CSL + 2.5% urea; C5U, 9% CSL + 5% urea.

Alpha diversity of attached bacteria on the surface of RS was shown by the Shannon or Chao1 index between different groups, and Chao1 results showed no significant differences in different groups at incubation time (Supplementary Figure 1A), while the Shannon index had slight increases in the CU group at 24 h (Supplementary Figure 1B). Diverse microbial compositions were detected in different groups at family levels (Supplementary Figure 2). Principal coordinate analysis (PCoA) based on weighted UniFrac distance indicated dispersed data points on plots of different groups (Supplementary Figure 3), implying the attached bacteria of RS affected by CSL and urea pre-treatment. Although the Chao1 index was similar in all four groups, obvious alternations of the microbial structure were detected. Notably, LEfSe analysis showed that *Aerococcaceae, Enterococcaceae, Carnobacteriaceae, Staphylococcaceae, Aerococcus, Enterococcus, Carnobacterium*, and *Staphylococcus* were enriched post-CSL and urea pre-treatment (Figure 5). In fact, the types of bacteria colonized on straw surfaces are closely related to degradation (Wright and Klieve, 2011). In this study, we found the *Clostridium_sensu_stricto_12, norank_f__norank_o__WCHB1_41, Atopobium*, and *Candidatus_Saccharimonas* enriched in the Con group; these bacteria had a negative for fiber degraded (Alonso et al., 2015). On the contrary, our results confirm previous reports that the *Carnobacterium, Atopobium*, and *Staphylococcaceae* were positive for fiber degraded (Xia et al., 2017; Tian et al., 2019). Interestingly, the *Carnobacteriaceae, Staphylococcaceae, Aerococcus, Carnobacterium*, and *Staphylococcus* were always attached to the CU and C5U during the entire incubation process in the rumen. We speculated that may be the role played by CSL because no adhesion of these bacteria was found in the U.

### The Link Between Rumen Bacterial Attachment to the Surface of RS and *In vitro* Degradability

**Figure 6:**
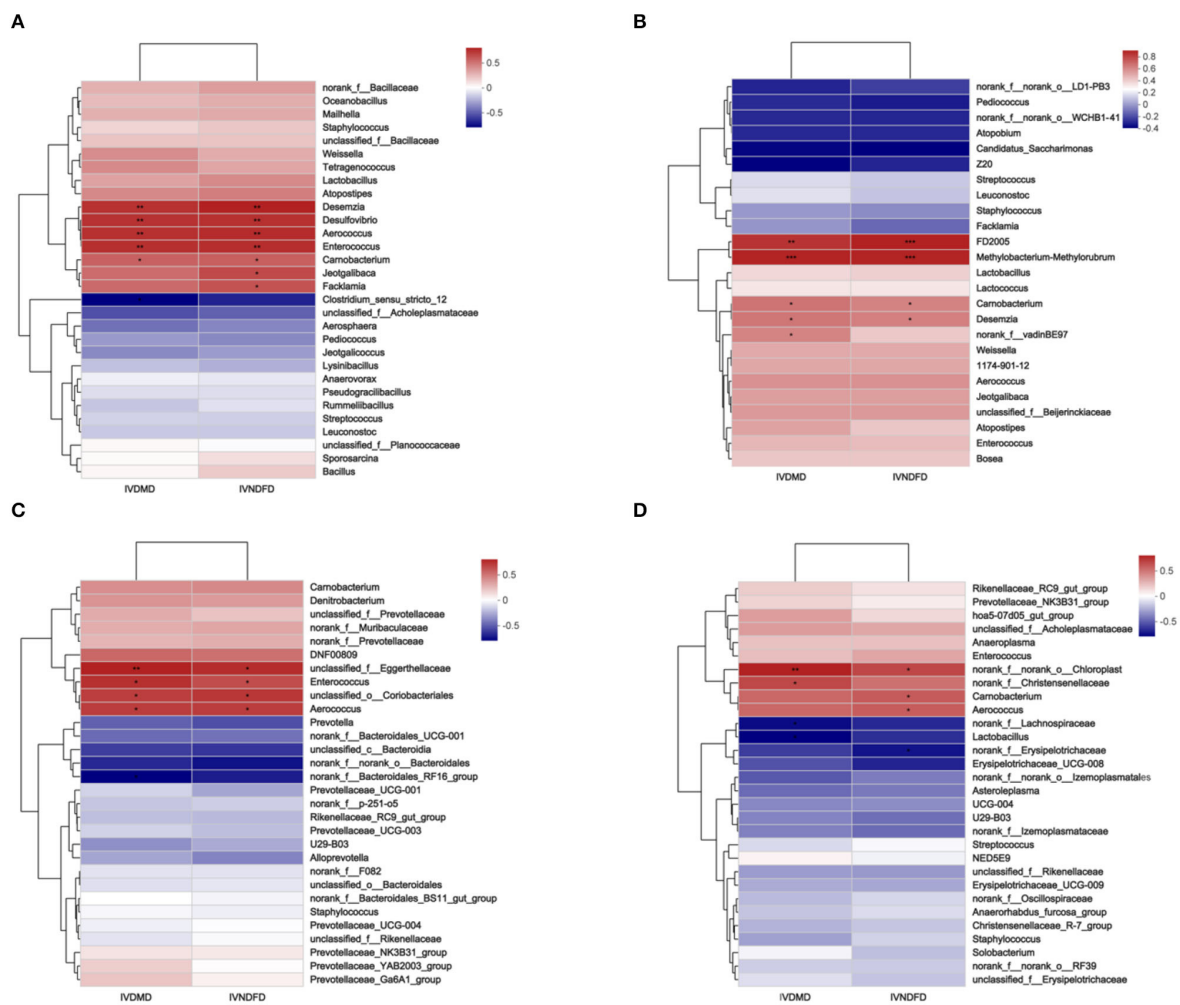
Correlations among the colonization microbial on surface of RS samples after incubation 0.5 h **(A)**, 4 h **(B)**, 12 h **(C)**, and 24 h **(D)** and *in vitro* degradability [our previously published research (Ma et al., 2020)]. Con, without additive control; U, 5% urea; CU, 9% CSL + 2.5% urea; C5U, 9% CSL + 5% urea. *, **, and *** indicate the significant correlations at *P* < 0.05, 0.01, and 0.001. IVDMD, *in vitro* dry matter degradability, IVNDFD, *in vitro* neutral detergent fiber degradability.

The relationship between rumen bacterial attachment on RS surface and *in vitro* degradability (IVDMD and IVNDFD) and these results had been published by us (Ma et al., 2020). As shown in Figures 6A,D, the *Aerococcus* (*P* < 0.001) and *Carnobacterium* (*P* < 0.05) had significantly positive repercussions on IVDMD and IVNDFD of RS after incubating in the rumen at 0.5, 4, and 12 h (Figures 6A–C), while *Carnobacterium* and *Aerococcus* had a significant positive effect on IVNDFD (*P* < 0.05) of RS after incubation for 24 h in the rumen (Figure 6D). Notably, *Carnobacterium* and *Aerococcus* were enriched in CU and C5U groups, which might be caused by CSL inducing the colonization of these two bacteria on the surface of RS since this phenomenon was not observed in U and Con groups. Importantly, *Carnobacterium* and *Aerococcus* had a strong fiber degradation ability (Xia et al., 2017; Jo et al., 2021). The *norank_f__norank_o__WCHB1_41, Atopobium*, and *Candidatus_Saccharimonas* had a negative impact on the IVDMD and IVNDFD of RS in Con after 4 h of incubation in the rumen (Figures 6A–D).

## Conclusion

The CSL and urea pre-treatment significantly reduced NDF and ADF contents and increased RS's CP content and WSC yield. The CSL and urea pre-treatment leads to enhanced microbial colonization by destructing the RS structure. Notably, CSL could induce the *Aerococcus, Carnobacterium*, and *Staphylococcus* attachment on the surface of RS. These findings will facilitate the further application of CSL and urea in the degradation of lignocellulosic biomass.

## Data Availability Statement

The original contributions presented in the study are included in the article/Supplementary Material, further inquiries can be directed to the corresponding author/s.

## Ethics Statement

The *in situ* experimental procedure was approved by the Ethical Committee of the College of Animal Science and Technology of China Agricultural University (Protocol number: 2013-5-LZ).

## Author Contributions

ZC, YM, and XC mainly designed this experiment. YM conducted the animal experiment and collected and analyzed the data. YM mainly wrote the manuscript and XC, MK, JX, GA, SL, JW, and ZC edited it. All authors contributed to the article and approved the submitted version.

## Funding

This study was supported by the 2115 Talent Development Program of China Agricultural University.

## Conflict of Interest

The authors declare that the research was conducted in the absence of any commercial or financial relationships that could be construed as a potential conflict of interest.

## Publisher's Note

All claims expressed in this article are solely those of the authors and do not necessarily represent those of their affiliated organizations, or those of the publisher, the editors and the reviewers. Any product that may be evaluated in this article, or claim that may be made by its manufacturer, is not guaranteed or endorsed by the publisher.
