# Supplementary material for: Effect of the Combining Corn Steep Liquor and Urea Pre-treatment on Biodegradation and Hydrolysis of Rice Straw
Source: Front Microbiol. 2022 Jul 13;13:916195. doi: 10.3389/fmicb.2022.916195 (PMC9326473; doi:10.3389/fmicb.2022.916195)
Supplement: Supplementary file 1 [file Data_Sheet_1.PDF]

Table S1

Supplemental Table 1. Basic diet composition and nutrient value (DM basis %)

Items	Content	Nutrient levels[2]	Content
Ingredients		NEL (MJ/Kg)	5.68
Chinese wildrye	6.38	CP	15.01
Alfalfa hay	20.31	EE	3.4
Oat hay	5.58	NDF	41.03
Wheat	1.76	ADF	26.69
Corn silage	24.48	Ca	0.55
Corn	3.66	P	0.39
Steam-flaked corn	13.52		
Soybean meal	6.55		
Extruded soybean	3.76		
Soybean hull	3.00		
Cottonseed	3.41		
Molasses	3.80		
Rumen-pass fatty acid	1.20		
Yeast powder	0.20		
Mycotoxin removement agent	0.06		
NaCL	0.31		
Limestone	0.32		
$Ca(HCO_3)_2$	0.34		
$NaHPO_3$	0.67		
$KHCO_3$	0.26		
Premix[1]	0.31		
MgO	0.12		

1) Each kilogram of premix contains VA 1 000 000 IU, VD3 280 000 IU, VE 10 000 IU, nicotinic acid 1 000 mg, Cu (as copper sulfate) 3 250 mg, Mn 4 800 mg, Zn 12 850 mg, I 140 mg, Se 150 mg, Co 110 mg.

2) NEL was a calculated value, while the other nutrient levels were measured values.

Figure S1

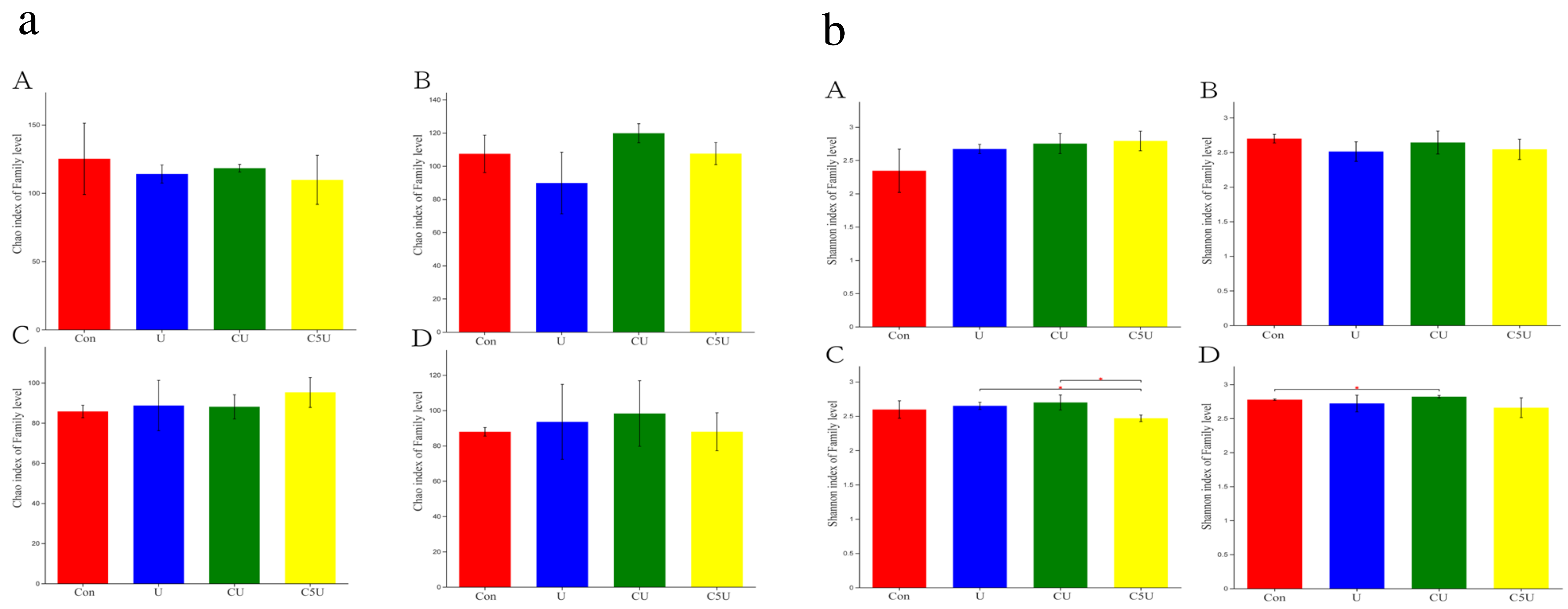

a
A
Chao index of Family level
Con
U
CU
C5U
B
Chao index of Family level
Con
U
CU
C5U
C
Chao index of Family level
Con
U
CU
C5U
D
Chao index of Family level
Con
U
CU
C5U
b
A
Shannon index of Family level
Con
U
CU
C5U
B
Shannon index of Family level
Con
U
CU
C5U
C
Shannon index of Family level
Con
U
CU
C5U
D
Shannon index of Family level
Con
U
CU
C5U

Figure S2

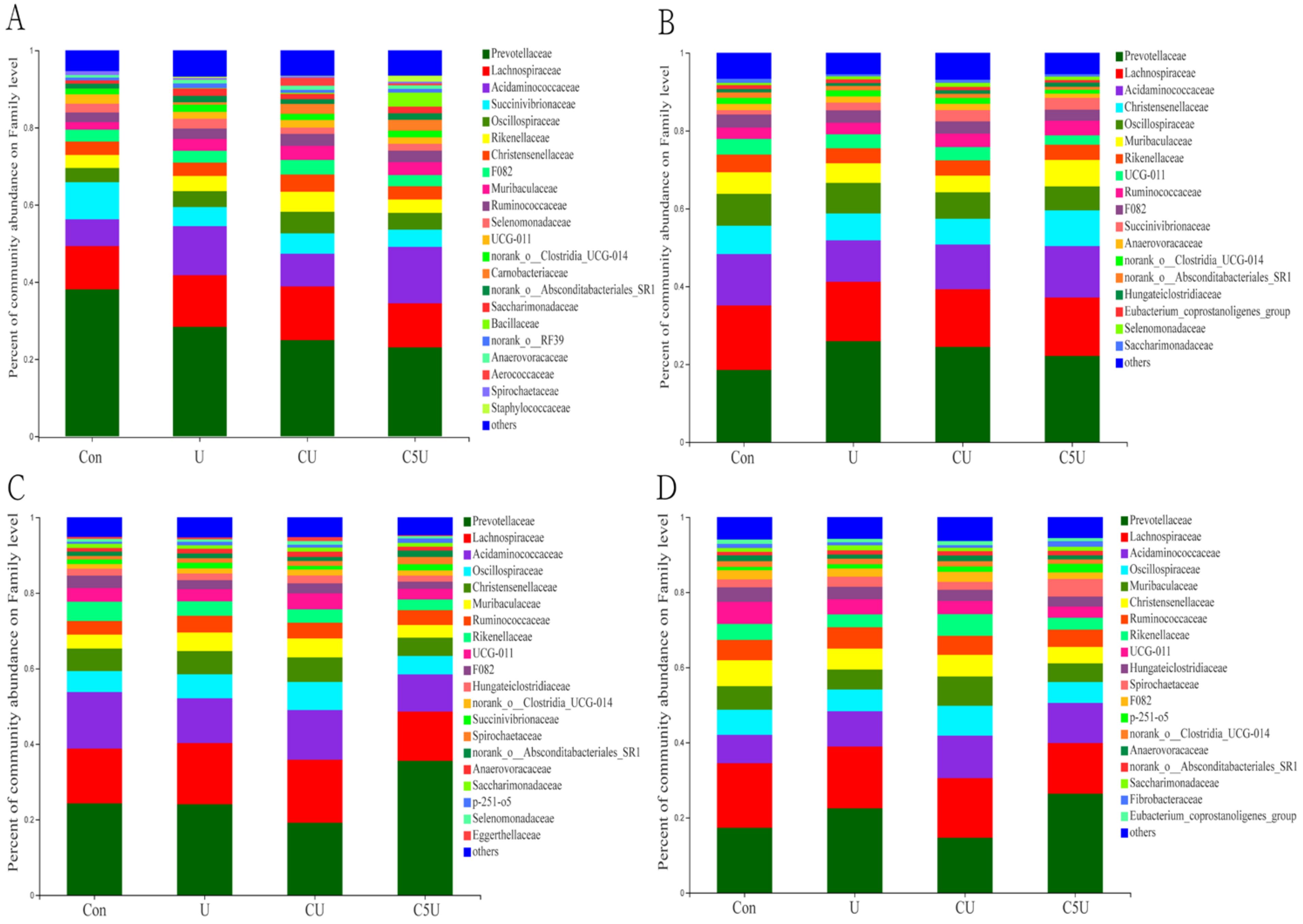
A
Percent of community abundance on Family level
0
0.2
0.4
0.6
0.8
1
Con
U
CU
C5U
Prevotellaceae
Lachnospiraceae
Acidaminococcaceae
Succinivibrionaceae
Oscillospiraceae
Rikenellaceae
Christensenellaceae
F082
Muribaculaceae
Ruminococcaceae
Selenomonadaceae
UCG-011
norank_o__Clostridia_UCG-014
Carnobacteriaceae
norank_o__Absconditabacteriales_SR1
Saccharimonadaceae
Bacillaceae
norank_o__RF39
Anaerovoracaceae
Aerococcaceae
Spirochaetaceae
Staphylococcaceae
others
B
Percent of community abundance on Family level
0
0.2
0.4
0.6
0.8
1
Con
U
CU
C5U
Prevotellaceae
Lachnospiraceae
Acidaminococcaceae
Christensenellaceae
Oscillospiraceae
Muribaculaceae
Rikenellaceae
UCG-011
Ruminococcaceae
F082
Succinivibrionaceae
Anaerovoracaceae
norank_o__Clostridia_UCG-014
norank_o__Absconditabacteriales_SR1
Hungateiclostridiaceae
Eubacterium_coprostanoligenes_group
Selenomonadaceae
Saccharimonadaceae
others
C
Percent of community abundance on Family level
0
0.2
0.4
0.6
0.8
1
Con
U
CU
C5U
Prevotellaceae
Lachnospiraceae
Acidaminococcaceae
Oscillospiraceae
Christensenellaceae
Muribaculaceae
Ruminococcaceae
Rikenellaceae
UCG-011
F082
Hungateiclostridiaceae
norank_o__Clostridia_UCG-014
Succinivibrionaceae
Spirochaetaceae
norank_o__Absconditabacteriales_SR1
Anaerovoracaceae
Saccharimonadaceae
p-251-o5
Selenomonadaceae
Eggerthellaceae
others
D
Percent of community abundance on Family level
0
0.2
0.4
0.6
0.8
1
Con
U
CU
C5U
Prevotellaceae
Lachnospiraceae
Acidaminococcaceae
Oscillospiraceae
Muribaculaceae
Christensenellaceae
Ruminococcaceae
Rikenellaceae
UCG-011
Hungateiclostridiaceae
Spirochaetaceae
F082
p-251-o5
norank_o__Clostridia_UCG-014
Anaerovoracaceae
norank_o__Absconditabacteriales_SR1
Saccharimonadaceae
Fibrobacteraceae
Eubacterium_coprostanoligenes_group
others

Figure S3

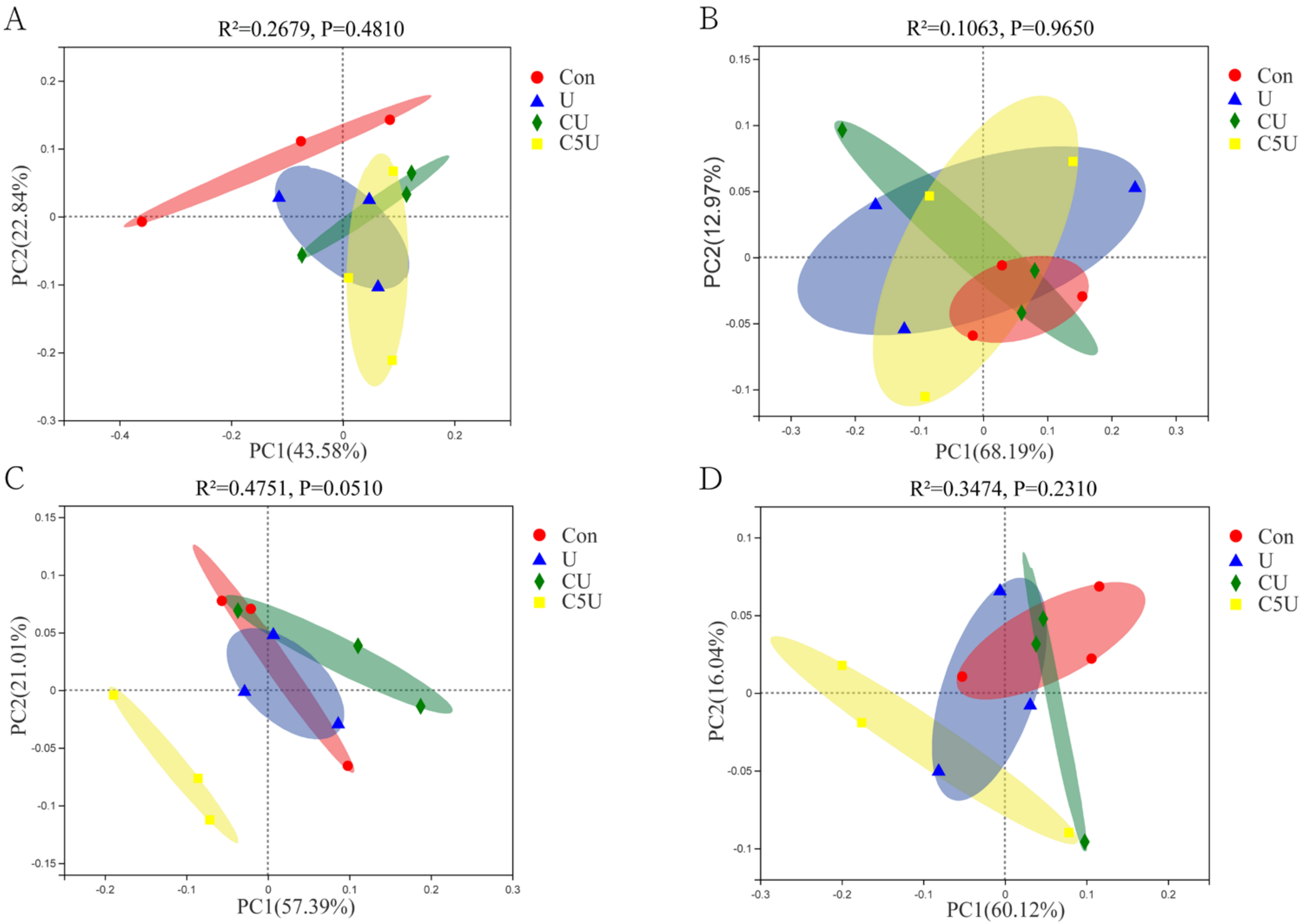
A
R²=0.2679, P=0.4810
PC1(43.58%)
PC2(22.84%)
Con
U
CU
C5U
B
R²=0.1063, P=0.9650
PC1(68.19%)
PC2(12.97%)
Con
U
CU
C5U
C
R²=0.4751, P=0.0510
PC1(57.39%)
PC2(21.01%)
Con
U
CU
C5U
D
R²=0.3474, P=0.2310
PC1(60.12%)
PC2(16.04%)
Con
U
CU
C5U
