# Supplementary material for: Effect of the Combining Corn Steep Liquor and Urea Pre-treatment on Biodegradation and Hydrolysis of Rice Straw
Source: Front Microbiol. 2022 Jul 13;13:916195. doi: 10.3389/fmicb.2022.916195 (PMC9326473; doi:10.3389/fmicb.2022.916195)
Supplement: Supplementary file 2 [file Data_Sheet_2.PDF]

Effect of the combining corn steep liquor and urea-pretreatment on biodegradation and hydrolysis of rice straw

Yulin Ma[a], Xu Chen[a], Muhammad Zahoor Khan[a], Jianxin Xiao[a], Gibson Maswayi Alugongo[a], Shuai Liu[a], Jingjun Wang[a], Zhijun Cao[a]*

[a]*State Key Laboratory of Animal Nutrition, Beijing Engineering Technology Research Center of Raw Milk Quality and Safety Control, College of Animal Science and Technology, China Agricultural University, Beijing 100193, PR China;*

**Correspondence: caozhijun@cau.edu.cn; Tel.: +86-10-62733746*

Supplementary Information

Figure S1. α-diversity of different groups after incubation in rumen 0.5 h (A), 4 h (B), 12 h (C) and 24 h (D) by chao1(a) and Shannon (b) index. Con: without additive control, U: 5% urea, CU: 9% CSL + 2.5% urea, C5U: 9% CSL+ 5% urea. Data were mean ± SEM. *P* values were determined using the t-test. **P*≤0.05, ***P*≤0.01.

Figure S2. The relative abundance of colonization bacterial on surface of rice straw of different groups after incubation in rumen 0.5 h (A), 4 h (B), 12 h (C) and 24 h (D) at family level presented in 99.5% of the community. Con: without additive control, U: 5% urea, CU: 9% CSL + 2.5% urea, C5U: 9% CSL+ 5% urea.

Figure S3. PCoA plots based on the weighted UniFrac distance matrix upon different groups after incubation in rumen 0.5 h (A), 4 h (B), 12 h (C) and 24 h (D). The data were assessed using PERMANOVA analysis, with 999 permutations. Con: without additive control, U: 5% urea, CU: 9% CSL + 2.5% urea, C5U: 9% CSL+ 5% urea.
